# Other PHAC publications

**DOI:** 10.24095/hpcdp.44.10.06

**Published:** 2024-10

**Researchers from the Public Health Agency of Canada also contribute to work published in other journals and books. Look for the following articles published in 2024:**

**Alami A, Dave S, Uhlik C, Ebrahim M**, Krewski D, **Laroche J**. Determinants of influenza non-vaccination among Canadian children: insights from a nationwide survey. Front Public Health. 2024;12:1400782. https://doi.org/10.3389/fpubh.2024.1400782

Buxton RT, Hudgins EJ, Lavigne E, Villeneuve PJ, **Prince SA**, Pearson AL, et al. Mental health is positively associated with biodiversity in Canadian cities. Commun Earth Env. 2024;5(1):310. https://doi.org/10.1038/s43247-024-01482-9

Chen KY, Eddeen AB, Bennett C, **Yusuf W**, Hennessy D, Barnes JD, et al. Trends in cardiovascular risk factors in Canada: variation by migration and temporal factors, 2001-2018. CJC Open. 2024;6(8):951-8. https://doi.org/10.1016/j.cjco.2024.04.006

Karmali S, **Saxena S, Richards O, Thompson W, McFaull SR**, Pike I. What was the impact of COVID-19 restrictions on unintentional injuries, in Canada and globally? A scoping review investigating how lockdown measures impacted the global burden of unintentional injury. Front Public Health. 2024;12:1385452. https://doi.org/10.3389/fpubh.2024.1385452

Maral IR, Vidal-Almela S, Blanchard C, **Prince SA**, Way KL, Reed JL. Sex differences in physical activity levels and sitting time in patients with atrial fibrillation. J Cardiopulm Rehabil Prev. 2024;4:280-8. https://doi.org/10.1097/HCR.0000000000000867

**Moqueet N**, Cornacchi SD, Antony J, Khalil I, Manca D, Fernandes C, et al. BETTER LIFE- guidelines for chronic disease preventive care for people aged 18–39 years: a literature review. BMC Prim Care. 2024;25(1):224. https://doi.org/10.1186/s12875-024-02471-9

**Prince SA**, Biswas A, **Betancourt MT, Toigo S, Roberts KC**, Colley RC, […] **Lang JJ**. Telework and 24-hour movement behaviours among adults living in Canada during the COVID-19 pandemic. Prev Med. 2024;185:108053. https://doi.org/10.1016/j.ypmed.2024.108053

**Rakotovao L**, Simeoni M, Bennett-AbuAyyash C, Walji T, Abdi S. Addressing anti-Black racism within public health in North America: a scoping review. Int J Equity Health. 2024;23(1):128. https://doi.org/10.1186/s12939-024-02124-4

Ukah UV, Ct-Corriveau G, **Nelson C**, Healy-Profits J, Auger N. Risk of adverse neonatal events in pregnancies complicated by severe maternal morbidity. J Pediatr. 2024;273:114149. https://doi.org/10.1016/j.jpeds.2024.114149

Villeneuve PJ, **Morrison HI**, Lane R. Updated analysis of radon exposure and lung cancer mortality in the cohort of Newfoundland fluorspar miners (1950–2016). Radiat Res. 2024;202(1):59-69. https://doi.org/10.1667/RADE-23-00114.1

**Zuckermann AM, Morissette K, Boland L, Garcia AJ, Domingo FR**, Stockwell T, et al. The effects of alcohol container labels on consumption behaviour, knowledge, and support for labelling: a systematic review. Lancet Public Health. 2024;7:e481-94. https://doi.org/10.1016/S2468-2667(24)00097-5

